# Preliminary Evidence on the Diagnostic and Molecular Role of Circulating Soluble EGFR in Non-Small Cell Lung Cancer

**DOI:** 10.3390/ijms160819612

**Published:** 2015-08-19

**Authors:** Filippo Lococo, Massimiliano Paci, Cristian Rapicetta, Teresa Rossi, Valentina Sancisi, Luca Braglia, Silvio Cavuto, Alessandra Bisagni, Italia Bongarzone, Douglas M. Noonan, Adriana Albini, Sally Maramotti

**Affiliations:** 1 Thoracic Surgery Unit, Department of Cardiology, Thoracic and Vascular Surgery, IRCCS Arcispedale Santa Maria Nuova, Reggio Emilia 42123, Italy; E-Mails: filippo.lococo@asmn.re.it (F.L.); massimiliano.paci@asmn.re.it (M.P.); cristian.rapicetta@asmn.re.it (C.R.); 2 Laboratory of Translational Research, Research and Statistic Infrastructure, IRCCS Arcispedale Santa Maria Nuova, Reggio Emilia 42123, Italy; E-Mails: teresa.rossi@asmn.re.it (T.R.); valentina.sancisi@asmn.re.it (V.S.); sally.maramotti@asmn.re.it (S.M.); 3 Clinical Trials and Statistics Unit, Department of Infrastructure Research and Statistics, IRCCS Arcispedale Santa Maria Nuova, Reggio Emilia 42123, Italy; E-Mails: luca.braglia@asmn.re.it (L.B.); silvio.cavuto@asmn.re.it (S.C.); 4 Pathology Unit, Department of Oncology and Advanced Technologies, IRCCS Arcispedale Santa Maria Nuova, Reggio Emilia 42123, Italy; E-Mail: alessandra.bisagni@asmn.re.it; 5 Proteomics Laboratory, Department of Experimental Oncology and Molecular Medicine, Fondazione IRCCS Istituto Nazionale Tumori, Milan 20133, Italy; E-Mail: italia.bongarzone@istitutotumori.mi.it; 6 Department of Biotechnology and Life Sciences, University of Insubria, Varese 21100, Italy; E-Mail: douglas.noonan@uninsubria.it; 7 Scientific and Technology Park, IRCCS MultiMedica, Milan 20138, Italy

**Keywords:** Epidermal growth factor receptor (EGFR), EGFR isoform (sEGFR), non-small cell lung cancer (NSCLC), biomarker, diagnosis

## Abstract

Assessment of biological diagnostic factors providing clinically-relevant information to guide physician decision-making are still needed for diseases with poor outcomes, such as non-small cell lung cancer (NSCLC). Epidermal growth factor receptor (EGFR) is a promising molecule in the clinical management of NSCLC. While the EGFR transmembrane form has been extensively investigated in large clinical trials, the soluble, circulating EGFR isoform (sEGFR), which may have a potential clinical use, has rarely been considered. This study investigates the use of sEGFR as a potential diagnostic biomarker for NSCLC and also characterizes the biological function of sEGFR to clarify the molecular mechanisms involved in the course of action of this protein. Plasma sEGFR levels from a heterogeneous cohort of 37 non-advanced NSCLC patients and 54 healthy subjects were analyzed by using an enzyme-linked immunosorbent assay. The biological function of sEGFR was analyzed *in vitro* using NSCLC cell lines, investigating effects on cell proliferation and migration. We found that plasma sEGFR was significantly decreased in the NSCLC patient group as compared to the control group (median value: 48.6 *vs.* 55.6 ng/mL respectively; *p* = 0.0002). Moreover, we demonstrated that sEGFR inhibits growth and migration of NSCLC cells *in vitro* through molecular mechanisms that included perturbation of EGF/EGFR cell signaling and holoreceptor internalization. These data show that sEGFR is a potential circulating biomarker with a physiological protective role, providing a first approach to the functional role of the soluble isoform of EGFR. However, the impact of these data on daily clinical practice needs to be further investigated in larger prospective studies.

## 1. Introduction

Primary lung cancer is the most common malignancy and the leading cause of human cancer deaths worldwide [1]. Even though many efforts to improve lung cancer outcomes have been accomplished, long-term survival has not improved significantly over the last 20 years, with a five-year cumulative survival rate that remains at only 15%. The role of early diagnosis using low-dose computed tomography (LDCT) in lung cancer mortality reduction is questioned by many researchers [2], despite the results of National Lung Screening Trial Research Team (NLST), which provided important information regarding the use of CT to reduce lung cancer mortality [3]. On the other hand, to date, there is no consensus on the proven clinical value for use of biomarkers for diagnosis, staging, prognosis, and monitoring for disease relapse or treatment response in lung cancer. The development of biomarkers for lung cancer is a public health imperative since accurate diagnosis and treatment are associated with up to a 92% five-year survival [4]. Epidermal Growth Factor Receptor (EGFR) is a membrane-bound tyrosine kinase glycoprotein, expressed on the surface of epithelial cells [5]. It has been implicated in the molecular pathogenesis of several cancers, including non-small cell lung cancer (NSCLC) [6]. EGFR is composed of an extracellular domain (ECD), a transmembrane lipophilic segment, and an intracellular domain (ICD) containing a tyrosine kinase domain. EGFR is best known for its classical function as a receptor tyrosine kinase localized on the plasma membrane and activated upon ligand binding. Activated EGFR recruits a number of downstream signaling molecules, leading to the activation of several pathways involved in tumor growth, progression, and survival [7]. In addition to the full-length transmembrane form (p170) of EGFR (Figure S1, Isoform A), normal and malignant cells synthesize soluble EGFR isoforms (sEGFRs) that embody extensive portions of the ECD. These isoforms are produced either by proteolytic cleavage of the full-length receptor or by alternative splicing of mRNA transcripts, but their biological functions remain unclear [8,9]. The existence of two highly-glycosylated EGFR soluble isoforms of about 110 kDa each, composed of the ECD of the receptor, has been reported [8,10]. The isoform B (Figure S1) arises by proteolytic cleavage of EGFR at the plasma membrane between the amino acid residues gly 625 and met 626. This isoform is called PI-sEGFR and was detected in the conditioned medium of malignant cell lines that express 7 × 10^5^ or higher receptors/cell [8]. The isoform C (Figure S1), also called p110 sEGFR, is derived from a 3-kb alternate mRNA transcript of the human EGFR protoncogene [10]. This protein corresponds to the extracellular domain of EGFR to amino acid residue 603, followed by 78 unique COOH-terminal amino acids. The p110 EGFR is associated with the cell membrane [11] and can be released, allowing detection in human serum and plasma [9,12]. The existence of this circulating sEGFR isoform stimulated investigation of its role as a potential circulating biomarker. Several studies suggest that alterations in sEGFR levels may be useful in cancer diagnosis and in monitoring disease recurrence and outcome, especially in patients with ovarian or breast cancer [9,13,14,15]. In the context of lung cancer, the debate about the use of sEGFR for NSCLC diagnosis is ongoing, although sEGFR has been studied in predicting prognosis and therapeutic responsiveness [16,17]. A few preliminary studies were conducted and the data obtained remain unclear; these publications report the puzzling observation that circulating sEGFR levels were higher in healthy subjects and tended to decrease in patients with neoplastic lesions [18,19,20]. Moreover, the biological role of sEGFR and the course of action of this protein in the context of NSCLC still needs to be defined. Here we investigated whether sEGFR may be a potential biomarker able to predict diagnosis in NSCLC patients, showing a significant negative correlation between NSCLC and sEGFR levels. We further characterized the biological function of sEGFR using *in vitro* models, demonstrating the antitumor activity of sEGFR and providing a first approach towards characterizing the functional role of the soluble isoform of EGFR.

## 2. Results

Patients (*n* = 37) and controls (*n* = 54) exhibit baseline differences regarding age (mean ± SD are 69.9 ± 8.3 for patients, 58.6 ± 6.0 for controls, *p* < 0.0001) and smoking habits (smokers are 81% of patients, 20.4% of controls, *p* < 0.0001). Gender distribution is not statistically different (females are 29.7% of patients, 18.5% for controls, *p* = 0.320). Focusing on cases, surgical procedures consisted of (bi)lobectomy in 32 cases (86.5%) and anatomic segmentectomy in the remaining five cases (13.5%), while no patients underwent pneumonectomy. Radical lymphnodal dissection was performed in all but two patients (who underwent mediastinal nodal sampling). Complete resection was achieved in all cases. Histological examination revealed 27 adenocarcinomas and 10 squamous cell carcinomas.

### 2.1. Plasma Levels of sEGFR Are Lower in NSCLC Patients than Healthy Controls

**Figure 1:**
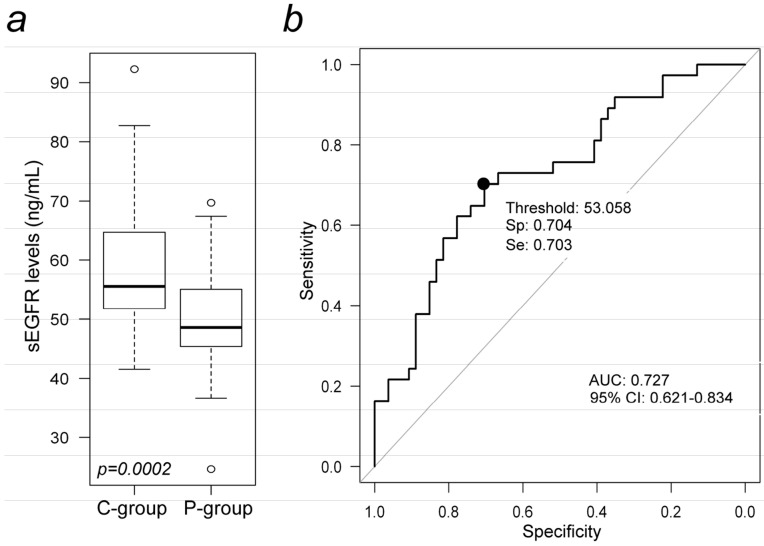
Quantification and diagnostic accuracy analysis of sEGFR levels in plasma of NSCLC patients and healthy controls. (**a**) Box plot illustrate the distributions of sEGFR (ng/mL) levels in plasma from 37 NSCLC patients (P-group) and 54 healthy controls (C-group). Horizontal bold line, median value; the interquartile range is indicated above and below each bar; circles, outliers; (**b**) The receiver operating characteristic (ROC) curves of plasma sEGFR in discriminating NSCLC patients from healthy subjects. The area under the ROC curves (AUC) was 0.727 (95% CI: 0.621 to 0.834). The point on the curve indicates the cut-off value (53.058 ng/mL), for which sensitivity and specificity were 70.4% and 70.3%, respectively. The odds ratio according to the cut-off value was 5.613 (95% CI: 2.247 to 14.023).

Levels of sEGFR were examined by ELISA in a total of 91 plasma samples, 37 from patients with NSCLC and 54 healthy subjects. The levels of plasma sEGFR were significantly lower in patients with NSCLC as compared with healthy controls (median values: 48.6 *vs.* 55.6 ng/mL respectively; *p* = 0.0002; Table 1, Figure 1a). We estimated and plotted a ROC curve to assess the potential usefulness of plasma sEGFR as a non-invasive biomarker for the diagnosis of NSCLC. The ROC analyses revealed that plasma sEGFR levels were reasonably robust in discriminating patients with NSCLC from control subjects, with an AUC value of 0.727 (95% CI: 0.621 to 0.834) (Figure 1b). Using a cut-off value of 53.058 ng/mL, and considering a sEGFR value under this limit as predictive of disease (positive test), the sensitivity and specificity were 70.4% and 70.3%, respectively. The odds ratio according to the same cut-off value was 5.613 (95% CI: 2.247 to 14.023). However, the marker contribution to a diagnostic model that included age (in continuous), gender and smoking habits, was no longer statistically significant (OR for 1 unit increase of sEGFR to “case diagnosis” is 0.978, 95% CI: 0.909 to 1.046, *p* = 0.519). Furthermore, examining the associations between the sEGFR expression levels with the clinical-pathological characteristics of the NSCLC patients, we found no significant association for age, gender, smoking habit, and histological type (Table 2), suggesting that these factors did not influence sEGFR levels. There was a positive trend when we analyzed the correlation between grading and sEGFR levels; patients who presented with a G3-NSCLC had lower values of sEGFR (47.5 ng/mL) as compared to patients with G1/G2-NSCLC (56.2 and 54.2 ng/mL), although this difference did not reach statistical significance (*p* = 0.082). We also examined whether copy number changes (polysomy) involving the EGFR locus impacted on diagnosis when analyzed in the context of the sEGFR levels. EGFR copy numbers were tested in all patients by FISH analysis. When we compared sEGFR levels of patients with EGFR amplification (*n* = 7) to patients having wild type EGFR-copy numbers (*n* = 30), no significant association between the groups (median values: 51.35 *vs.* 47.65 ng/mL respectively; *p* = 0.293; Figure S2a) was found. Representative FISH images from NSCLC patients harboring EGFR copy number amplification (Figure S2b) and having EGFR-copy number wild type (Figure S2c) are shown in Figure S2.

**Table 1:** Comparison between soluble epidermal growth factor receptor (sEGFR) concentration in non-small cell lung cancer (NSCLC) patients and healthy subjects.

sEGFR (ng/mL)	**Groups**	***n***	**M ± SD**	**Me**	**Range**	**M-WU Text**
	P-group	37	49.9 ± 9.3	48.6	24.7–69.7	*p* = 0.0002
	C-group	54	58.2 ± 10.5	55.6	41.5–92.3	

sEGFR, soluble epidermal growth factor receptor; P-Group, C-Group, healthy subjects; M ± SD (standard deviation); Me, median; *n*, number of samples; M-WU test, Mann-Whitney *U* test.

**Table 2:** sEGFR concentration according with demographic, clinical, and/or pathological characteristics.

Clinical-Pathological Variables	sEGFR (ng/mL)	*p* Value
**Age**		0.306
Age ≤ 65 (*n* = 10)	52.5 ± 13.3	
Age > 65 (*n* = 27)	48.9 ± 7.4	
**Gender**		0.512
Male (*n* = 26)	49.2 ± 7.5	
Female (*n* = 11)	51.4 ± 12.9	
**Smoking Habits**		0.639
Smokers (*n* = 30)	49.5 ± 8.1	
Non-Smokers (*n* = 7)	51.4 ± 14.0	
**Tumor Stage(TNM)**		0.588
pI (*n* = 21)	49.4 ± 7.3	
pII (*n* = 5)	53.9 ± 9.8	
pIII (*n* = 11)	49.0 ± 12.4	
**Histologic cell type**		0.907
ADK (*n* = 27)	49.7 ± 10.0	
SCC (*n* = 10)	50.2 ± 7.4	
**Grading**		0.082
G1 (*n* = 3)	56.2 ± 9.7	
G2 (*n* = 9)	54.2 ± 10.7	
G3 (*n* = 25)	47.5 ± 8.1	

sEGFR, soluble epidermal growth factor receptor; ADK, adenocarcinomas; SCC, squamous cell carcinomas.

### 2.2. Plasma Levels of sEGFR and Prognosis in NSCLC Patients

The study population was followed-up for a long-term clinical-radiological surveillance (median time 41.83 months, IQR: 25.99–46.86) through record linkages to death certificate databases and oncological registries. The median progression-free survival (PFS) was 42.2 months (95% CI: 31.5–NA). The mean sEGFR value recorded in the entire population was 49.9 ± 9.3 ng/mL, ranging from 24.7 ng/mL to 69.7 ng/mL. After Cox modeling of the PFS, we found that sEGFR, processed as a continuous variable, did not show a statistically significant role as a prognostic factor; HR = 0.987 (95% CI: 0.943–1.033) and *p* = 0.572 (the results were similar when we adjusted for sex, age, and smoking habits, HR = 0.988, 95% CI: 0.940–1.038, *p*-value = 0.618). However, considering its trend towards significance, we attempted to dichotomize the sEGFR distribution to locate a cut-off value useful for prognostic aims in clinical practice. Unfortunately, the Martingale residuals against the sEGFR plot (graph not shown) suggest that such a cut-off does not exist at all. Furthermore, adopting the Contal-O’Quigley approach, the best candidate as a possible cut-off (sEGFR = 45.8) reached only 0.964 as the *p*-value adjusted for multiplicity (based on a univariate Cox model having the dichotomized sEGFR as a predictive variable for PFS), while *p*-value without adjustment was 0.218.

### 2.3. sEGFR Has Growth and Migration Inhibitory Effects in EGFR-Wild Type NSCLC Cell Lines

**Figure 2:**
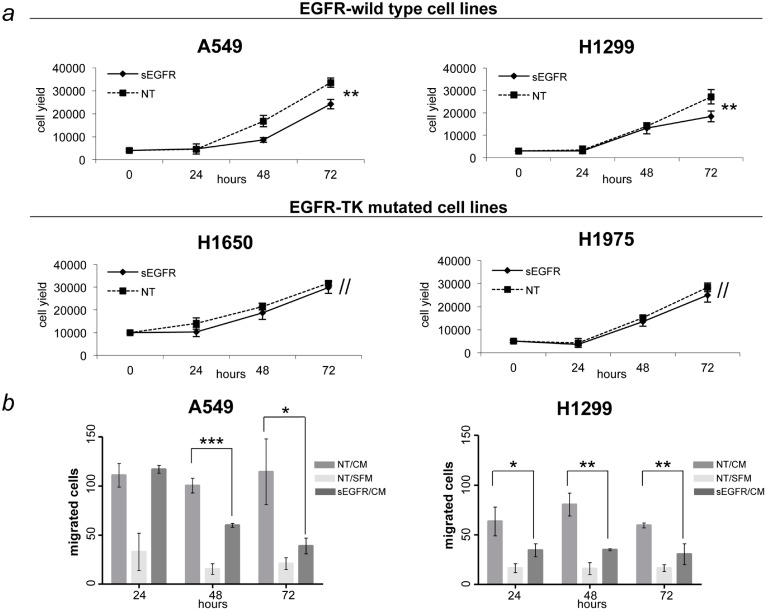
Effects of sEGFR on tumor cell growth and migration. (**a**) A549, H1299, H1650, and H1975 cell lines were exposed to sEGFR (1 µg/mL) or to the vehicle alone and cell counted over time; (**b**) A549 and H1299 cell lines were seeded into the upper compartment of Boyden chambers after 24, 48, and 72 h of pretreatment with sEGFR (1 µg/mL). Complete medium containing FBS 10% (CM) were placed in the lower compartment as a chemoattractant. After six hours of incubation, cells on the upper side of the filter were removed, and cells that migrated to the lower surface were fixed, stained, and counted. Serum-free medium (SFM) was used as negative control. Asterisks indicate significant differences (* *p* < 0.05, ** *p* < 0.01, *** *p* < 0.001, // *p* not significant) when comparing cells treated with sEGFR to those untreated (NT). Data are means ± SD from experiments with three replicates.

Since the plasma sEGFR levels were decreased in patients with NSCLC, we hypothesized that sEGFR may have a suppressive effect on NSCLC. To test this hypothesis, we investigated the molecular effect of sEGFRon NSCLC cells. Exposure to recombinant sEGFR of four NSCLC cell lines (A549, H1299, H1650, H1975) with different EGFR-mutation status characteristics were compared with untreated cells. In order to determine the best treatment-dose, we tested recombinant sEGFR on A549 cells at different doses ranging from 0.01 to 1 μg/mL. We chose 1 μg/mL as a test dose as it induced significant inhibition of cell proliferation without causing cellular toxicity (data not shown). Cell proliferation was evaluated after 24, 48, and 72 h by a cell counting assay. A different effect of sEGFR treatment was observed depending on the EGFR mutation status. The H1650 and H1975 cell lines, that contain activating mutations in EGFR, were not affected by the sEGFR treatment. In contrast, sEGFR treatment resulted in a significant inhibition of cell proliferation in the EGFR-wild type A549 and H1299 cell lines (Figure 2a). We excluded a contribution of endogenous sEGFR in the different cell response rates by performing ELISA assay on conditioned medium obtained from all cell lines (data not shown). To assess the effects of sEGFR on cell migration, A549 and H1299 cell lines treated or untreated with sEGFR for 24, 48, and 72 h were seeded into Boyden chambers and migrating cells were counted after six hours. sEGFR treatment resulted in a strong inhibition of cell migration in both wild type cell lines (Figure 2b). While 24 h of sEGFR treatment was sufficient to induce repression of migration in H1299 cells, in A549 cell line a longer exposure to this factor was needed in order to observe the inhibitory effect. Taken together, these observations suggest that sEGFR protein is capable of inhibiting tumor cell proliferation and cell migration in NSCLC cell lines devoid of EGFR mutations in their TK domain.

### 2.4. sEGFR Interferes with the EGF Signaling

**Figure 3:**
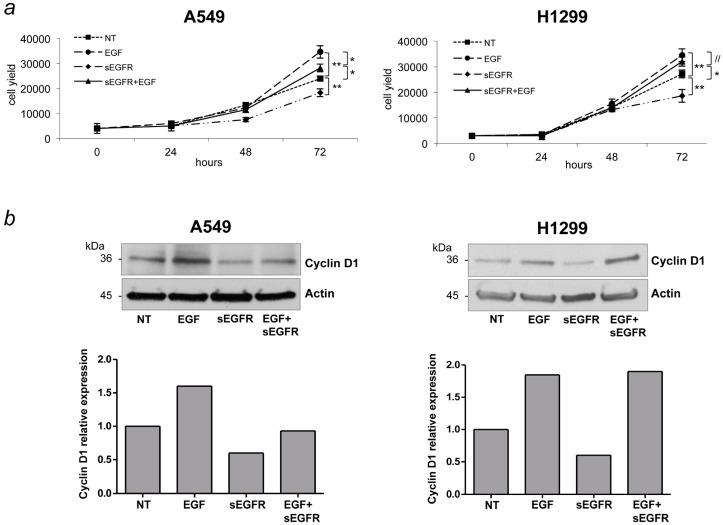
Effects of combined treatment with sEGFR and EGF on cell proliferation. EGFR-wild type cell lines (A549 and H1299) were treated with sEGFR (1 µg/mL) and EGF (100 ng/mL) alone and in combination (EGF + sEGFR). (**a**) Graph shows cell numbers at the indicated times after treatments. Untreated (NT) cells were used as a negative control. Asterisks indicate significant differences (* *p* < 0.05, ** *p* < 0.01, // *p* not significant) when comparing NT cells with: sEGFR, EGF, and sEGFR + EGF treatments. *p* value was also calculated for EGF-treated cells and those treated with EGF + sEGFR. Data are means ± SD from experiments with three replicates; (**b**) Western blot analysis of cyclin D1 expression levels in A549 and H1299 cell lysates obtained 24 h after treatment with sEGFR (1 ug/mL). β-actin was used as a loading control. The lower panels show cyclin D1 expression levels normalized to β-actin.

Soluble receptors exert their biological effects through different mechanisms of action [21]. They may play specific roles in modifying the biological response to ligands, interfering with the ligand-induced transduction signaling. Most commonly, soluble receptors compete with membrane-bound receptors for ligands in the extracellular milieu. This led us to hypothesize that the sEGFR protein may exert its activity by inhibiting the pro-oncogenic pathway induced by EGF. To characterize the sEGFR mechanism of action in NSCLC behavior, we investigated whether sEGFR could interfere with the EGF signaling. A549 and H1299 cell lines were treated with EGF recombinant protein in presence or absence of sEGFR. As expected, EGF treatment increased the cell proliferation in both cell lines (Figure 3a). Intriguingly, in A549 cells, the EGF proliferative effect was completely counteracted by the treatment with sEGFR. In contrast, in the presence of EGF, no significant effect of sEGFR was found in the H1299 cell line. The expression analysis of cyclin D1, a cell cycle marker whose down-regulation is consistent with cell cycle arrest, supported the proliferation data. We observed that cyclin D1 expression was up-regulated after EGF treatment and down-regulated after sEGFR treatment in both cell lines (Figure 3b). Consistent with the proliferation data, in A549 cells, sEGFR treatment restores basal levels of cyclin D1 expression in presence of EGF, while in H1299 cells the sEGFR treatment did not affect cyclin D1 expression in the presence of EGF. These data confirm that sEGFR protein inhibits NSCLC cell proliferation, likely by interfering with EGF signaling.

### 2.5. sEGFR Prevents the Plasma Membrane-EGFR Holoreceptor Internalization

**Figure 4:**
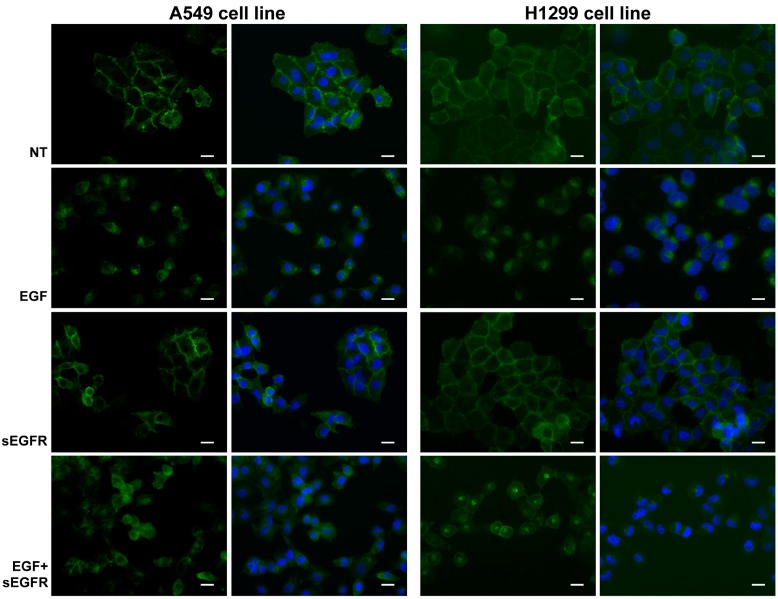
EGFR holoreceptor localization in lung tumor cells treated with sEGFR/EGF proteins. A549 and H1299 cell lines were untreated (NT) or treated with sEGFR (1 µg/mL) and EGF (100 ng/mL), alone and in combination (EGF + sEGFR), and EGFR holoreceptor localization was detected by using an immunofluorescent (IF) assay 24 h after treatment. Cells were treated with a polyclonal antibody against EGFR-ECD (Ab1_EGFR) and stained with FITC-conjugated anti-mouse secondary antibody (green). Cell nuclei were stained with DAPI (blue). Magnification: 40×. Scale bar: 20 µm.

To further understand the molecular mechanism of sEGFR on NSCLC cells, we analyzed the effect of the sEGFR treatment and its interaction with EGF protein on EGFR holoreceptor localization. A549 and H1299 cell lines were treated or not with sEGFR and EGF recombinant proteins, alone and in combination, and the EGFR localization was determined using an immunofluorescent (IF) assay. EGFR was mainly localized to the plasma membrane in both the cell lines before stimulation (Figure 4). After treatment with EGF, NSCLC cells showed a higher rate of receptor internalization, exhibiting perinuclear localization. A decrease in the EGFR levels was also observed. Treatment with sEGFR caused a clear EGFR localization and a homogeneous distribution at the membrane levels, preventing EGFR holoreceptor internalization. When the cells were treated with both EGF and sEGFR in combination, we observed a decrease in receptor internalization rate, with EGFR holoreceptor localized both at the membrane and cytoplasmic levels. We also observed a slight difference between A549 and H1299, in the H1299 cell line; the ratio between the internalized EGFR and plasma membrane EGFR levels was shifted toward the EGFR perinuclear localization. These data showed that the sEGFR affects EGFR holoreceptor extracellular domain internalization, thus interfering with EGF protein signaling.

### 2.6. EGF Treatment Inhibits sEGFR Synthesis in A549 Cells

**Figure 5:**
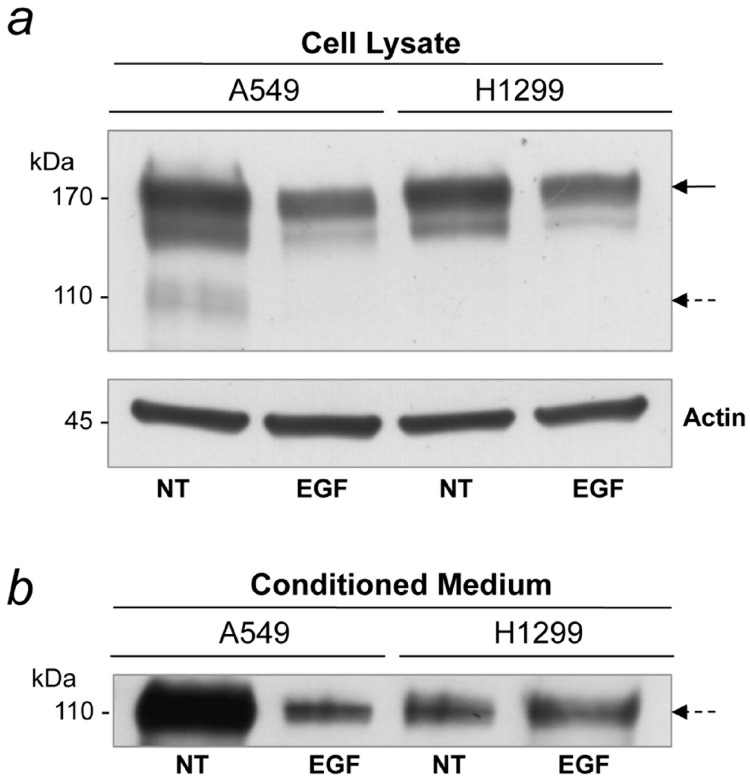
EGFR/sEGFR expression in tumor cells treated with EGF protein. A549 and H1299 cell lines were untreated or treated with EGF (100 ng/mL). (**a**) Western blot analysis of EGFR (170 kDa) and sEGFR (110 kDa) expression in A549 and H1299 cell lysates (50 µg) using the anti-EGFR ECD (Ab2_EGFR) antibody; (**b**) Western blot analysis of sEGFR (110 kDa) expression in A549 and H1299 conditioned medium (20 µg) using the anti-EGFR ECD (Ab2_EGFR) antibody. Solid arrows indicate the membrane p170 EGFR, and dashed arrows indicate the 110-kDa soluble EGFR.

Although sEGFR exhibited a strong inhibitory effect on the A549 and H1299 cell lines, some differences in the molecular response to sEGFR in the presence of EGF were observed. To test whether this discrepancy was due to intrinsic differences between the two cell types, A549 and H1299 cells were treated with recombinant EGF and compared with untreated cells. Cell lysate and conditioned medium were collected after 24 h of EGF treatment, and the sEGFR levels was detected by Western blot analysis. We previously showed that A549 cell line was able to produce a 110 kDa soluble isoform of EGFR. This isoform was detected in A549 cell line conditioned medium, had the same molecular weight of the plasma sEGFR, yet harbors different biochemical characteristics [20]. We observed that untreated cells from both lines were able to produce the sEGFR isoform (110 kDa), A549 and H1299 showed different levels of EGFR holoreceptor (170 kDa) (Figure 5a). The A549 cell line produced more sEGFR than the H1299 cell line, and this isoform was efficiently secreted into the medium (Figure 5b). Treatment with EGF caused a decrease in synthesis and secretion of sEGFR in the A549 cells, this effect was not observed in the H1299 cell line. These results indicate that sEGFR is a secreted protein produced by NSCLC cell lines whose production seems to be repressed by EGF stimulus in the A549 cell line.

## 3. Discussion

Assessment of biological diagnostic factors providing clinically-relevant information to guide physician decision-making are still needed for diseases with poor outcomes, such as NSCLC. The achievement of early diagnosis in NSCLC has been widely recognized to be crucial in the overall strategy of care and, accordingly, several screening program protocols adopting low-dose spiral computed tomography (LDCT) have been proposed to reduce overall mortality related with NSCLC. Nevertheless, the introduction of primary screening programs with LDCT has not been proposed on a large-scale population, to date; as stated by the NLST Research Team [22] “…recommendations on the basis of the findings of the NLST alone are insufficient to fully inform such important decisions”. Indeed, although the rate of death from any cause was reduced in the low-dose CT group, as compared with the radiography group, by 6.7% (95% CI, 1.2 to 13.6; *p* = 0.02) [3], before public policy recommendations are crafted, the cost-effectiveness of low-dose CT screening must be rigorously analyzed. The same NLST Research Team in the conclusion of the manuscript stated [22] that “…Other strategies for early detection of lung cancer, in particular, molecular markers in blood, sputum, and urine…- may one day help select persons who are best suited for low-dose CT screening or identify persons with positive low-dose CT screening tests who should undergo more rigorous diagnostic evaluation”. In this general scenario, it clearly emerged that the development of complementary strategies in the diagnostic process adopting bio-molecular markers stays as the main topic to be explored by the scientific community in the near future. EGFR is among the most promising molecules in the clinical management of this kind of tumor, considering its role in the carcinogenesis process and progression of NSCLC [6]. EGFR overexpression is observed in about 40%–80% of NSCLC patients and is related to a low grade of differentiation, increased tumor growth, and high metastatic rate [23,24]. Approved therapies targeting EGFR are now widely used for advanced NSCLC patients [25]. While the transmembrane EGFR form is well investigated in large clinical trials, the multiple isoforms of EGFR, that may have potential clinical use, are rarely considered. The isoforms containing only the ECD of the EGFR can be considered as “soluble receptors” since they can be detected in biological fluids such as serum and plasma. The biological function of these isoforms is not completely known. Baron *et al.* [9] were the first to identify a 110 kDa soluble isoform of EGFR (p110 sEGFR) in the bloodstream and to investigate the clinical utility of this circulating protein. Few studies have evaluated the role of sEGFR in NSCLC patients, with a dual aim of detecting its potential as a circulating tumor biomarker and its biological function. We previously characterized a 110 kDa sEGFR isoform with an extremely acidic isoelectric point (pI) (3.87–4.74) in plasma of NSCLC patients and healthy subjects. We demonstrated that this protein could discriminate between the presence or absence of lung cancer, despite the small number of subjects analyzed [20]. Here we extended these preliminary data, exploring potential of sEGFR as a diagnostic and/or prognostic biomarker in a larger and more heterogeneous cohort of non-advanced NSCLC patients. Regarding the diagnostic potential of sEGFR, we demonstrated that this protein could discern NSCLC patients from healthy subjects, showing a decrease in the patient group. Although the diagnostic value lost significance in multivariate analysis, univariate results are nonetheless meaningful considering the limited number of cases and the early phase of the study [26]. Further investigations including more homogeneous patient population should be designed to evaluate diagnostic performance. Decreased serum/plasma sEGFR has been reported both in lung cancer as compared to other neoplasms [9,18,19,20,27]. This observation is extremely puzzling and intriguing. If we consider that the term “circulating biomarker” means a molecule produced by the tumor and released in the bloodstream, the sEGFR may be defined as a “negative-biomarker”, that is, a physiological circulating molecule, probably produced by tissues that normally express the full-length EGFR and which levels decrease according with the tumor presence. When we analyzed the correlation between sEGFR and the pathological features of NSCLC patients, we found a positive trend between grading and sEGFR levels, indicating that lower values of sEGFR are detectable in patients with a high tumor grade, usually associated with clinical tumor progression and worse prognosis [28]. However, larger clinical cohorts are needed to consolidate this observation.

On the other hand, the data regarding the potential of sEGFR as a prognostic biomarker in patients with solid tumors from various sites are still conflicting. When considering NSCLC, only a few studies have been performed to evaluate if plasma sEGFR effectively influences the long-term survival [16,19]. Jantus-Lewintre and co-workers [16] reported, in a large cohort of advanced NSCLC patients, (308 patients with Stage IIIB/IV disease) a significant association between sEGFR value and prognosis, with lower baseline sEGFR values associated with reduced survival. Taking into proper consideration that our study was not sized to this aim, our findings showed just a non-statistical significant trend in line with the results obtained from Jantus-Lewintre *et al*. [16] (in terms of a protective effect of sEGFR), while we were not able to find a significant prognostic cut-off value in our cohort. The reasons may be that in our settings, the best candidate as possible cut-off (sEGFR = 45.8) is near the 32th percentile, while Jantus-Lewintre *et al*. [16] arbitrarily chose the third tercile.

The observation that plasma sEGFR levels decrease in NSCLC patients prompted us to investigate the biological function of this circulating protein *in vitro*. NSCLC cells frequently have activating mutations within the EGFR tyrosine kinase domain, which cause the constitutive activation of the EGFR-mediated signaling. Patients harboring these mutations are more sensitive to targeted therapies, such as gefitinib and erlotinib [29]. We, therefore, tested the effects of sEGFR in both TK-mutated and non-mutated NSCLC cell lines and we demonstrated that sEGFR showed anti-proliferative effects only in wild-type EGFR cell lines. We suggest a potential use of sEGFR in the clinical management of NSCLC patients not expressing EGFR TK mutations who are usually not responsive to EGFR target therapies. Restoring the physiological levels of circulating sEGFR in this group of patients might be an interesting therapeutic approach. The natural occurrence of circulating sEGFR produces yet another level of complexity on the regulation of EGFR receptor signal transduction.

To understand the biological role of sEGFR is of fundamental importance to clarify the course of action of this protein in the context of NSCLC, we therefore investigated the molecular mechanisms of sEGFR in NSCLC cells. The most known EGFR ligand is EGF, activating EGFR tyrosine kinase activity and downstream signaling pathways controlling cell proliferation, differentiation, survival, and motility [30]. Here we noted two major effects induced by sEGFR treatment in EGFR wild type NSCLC cell lines: the first was the decrease in cell proliferation and cyclin D1 expression, the second was the blocking of the EGFR holoreceptor internalization. Cyclin D1 plays a central role in the regulation of proliferation, linking the extracellular signaling environment to cell cycle progression, initiating transition from late G1 to the S phase of the cell cycle [31]. These data suggest that sEGFR inhibits the G1/S cell cycle transition. Moreover, there is emerging evidence that, when the EGFR holoreceptor is internalized, it translocates into the nucleus and acts as transcriptional factor, regulating the transactivation of the cyclin D1, iNOS, and Aurora-A together with the transcriptional factor STAT3 and STAT5 [32,33]. We observed that sEGFR treatment retained the EGFR holoreceptor at the plasma membrane, avoiding internalization, suggesting that EGFR interferes with EGF ligands and inhibits EGFR-nuclear translocation. Our data, obtained in wild type NSCLC cell lines treated with EGF, showed a perinuclear EGFR localization, further studies are warranted to investigate the presence of EGFR in the NSCLC cell nuclei. Interestingly, we noted slight differences in the responses to a combination of EGF and sEGFR. Treatment with EGF also induced a remarkable decrease in sEGFR production only in the A549 cell line. Moreover, the sEGFR basal levels produced by A549 and H1299 were notably different. We conclude that a relationship between sEGFR and EGF exists, and that the cellular response to sEGFR may be influenced by the basal levels of EGF produced.

In conclusion, these results suggest that sEGFR molecule harbors potential diagnostic value and provide an initial approach toward defining the functional role of sEGFR in NSCLC, demonstrating that sEGFR inhibits proliferation, migration, and perturbs the EGF/EGFR axis in NSCLC cell lines. Our data suggest that sEGFR may not only represent a novel potential biomarker for the NSCLC diagnosis but could potentially be a bio-therapeutic molecule.

## 4. Experimental Section

### 4.1. Patients and Control Subjects

Plasma samples from 37 patients with histopathologically-demonstrated NSCLC, and 54 healthy subjects, were prospectively collected at IRCCS-Santa Maria Nuova Hospital in Reggio Emilia (Italy), from August 2008 to March 2009. The study was conducted after the Ethical Committee approval and has therefore been performed in accordance with the ethical standards laid down in the 1964 Declaration of Helsinki and its later amendments. Written informed consent was obtained from all subjects before entering the study. Inclusion criteria were the presence of NSCLC ascertained with a cytohistological diagnosis and surgery with diagnostic and therapeutic aims. Controls come from healthy donors of a blood transfusion center. Patients and control subjects were not matched in this early phase investigation. Blood samples from both groups were obtained and stored under the same conditions. They were collected in test tubes containing K3-EDTA, and the plasma fraction was separated by two rounds of centrifugation at 2500 rpm for 10 min at 4 °C and then stored at −80 °C until analysis. Tumor specimens were routinely fixed in 4% buffered formalin and embedded in paraffin. Blood samples from patients were collected before surgery and none received neoadjuvant chemo and/or radiotherapy.

### 4.2. Clinical Data Collection and Statistical Analysis

Patients’ characteristics including age, gender, smoking habits, tumor stage, histologic cell type, and grading were recorded in a dedicated prospective database. Associations between the observation group (patients and controls) and common baseline variables (gender, smoking habits, and age) were assessed with two-tailed Chi-square and *t* tests. The sEGFR concentrations within patient and control groups were compared by two-tailed Mann-Whitney *U* test, while sEGFR concentration in patient groups according to demographic, clinical, and/or pathological characteristics were compared by ANOVA. Two-tailed Mann-Whitney *U* test was also used to compare sEGFR levels between patients harboring EGFR copy number amplification and those having EGFR-copy number wild type. Receiver operating characteristic (ROC) curve analysis was performed to assess the sEGFR diagnostic accuracy. The area under the ROC curve (AUC) was estimated with a 95% confidence interval (CI). Diagnostic cut-off was located by Youden-Index maximization; then sensitivity, specificity, and odds ratio were calculated. 95% CI for the odds ratio was calculated using the Wald method. To assess the relevance of sEGFR as a prognostic factor, it was processed as a continuous variable using the Cox approach in a model having the progression-free survival as the dependent variable. In order to explore whether sEGFR could be dichotomized, to provide an easily applicable cutoff for prognosis discrimination in clinical practice, Martingale residuals of a null model on PFS were plotted against sEGFR values (graph not shown). To the same aim, a possible cut-off was sought using the Contal-O’Quigley approach, with the accompanying multiplicity-adjusted *p*-value (based on a univariate Cox model having the dichotomized sEGFR as a predictive variable for PFS); the unadjusted *p*-value was provided as well. Proportional hazards assumptions for each Cox regression was tested assessing Schoenfeld residuals’ (null) slope against time (PH assumption was not refused in each one of the three Cox models, results not shown). Tests results were reported as *p*-values, and differences were considered statistically significant if *p* < 0.05. All statistical analyses were performed using SAS System release 9.2 (SAS Institute Inc., Cary, NC, USA) (descriptive statistics, ANOVA calculations, correlations), R release 3.1.1 (R Foundation for Statistical Computing, Vienna, Austria) (ROC and prognostic analysis) and GraphPad Prism release 5.0 (GraphPad Software, Inc., La Jolla, CA, USA) (remaining graphs).

### 4.3. Enzyme-Linked Immunosorbent Assay (ELISA)

Plasma sEGFR levels were assessed using a commercial Human EGFR Quantikine ELISA Kit (R&D Systems; Milan, Italy) and following the manufacturer’s procedures. The R&D ELISA kit utilizes a polyclonal antibody directed against the N-terminal portion of EGFR. A recombinant human EGFR (R&D Systems; Milan, Italy) was used as a positive control. Evaluation of intra- and inter-assay precision showed a coefficient of variation that was less than 5%.

### 4.4. Fluorescent in Situ Hybridization (FISH)

Formalin-fixed, paraffin-embedded tissue sections were stained with hematoxylin and eosin, and a quality control assessment of the tumor tissue was performed to ensure that sufficient material was available. FISH analysis was performed as previously described by Varella-Garcia and colleagues [34] by using the Vysis EGFR probe and a semi-automated procedure (Abbott Laboratories; Des Plaines, IL, USA). Tumors with four or more copies of the EGFR gene in ≥40% of the cells or tumors with EGFR gene amplification (gene-to-chromosome ratio ≥2 or presence of gene cluster or ≥15 gene copies in ≥10% of the cells) were considered to be FISH-positive, whereas all other tumors were considered to be FISH-negative. The same observer scored 50 tumor cells each in at least four tumor areas. All FISH analyses were performed in a blinded fashion without access to the patient clinical characteristics or treatment outcome.

### 4.5. Cell Culture Conditions, Cell Lysates and Conditioned Medium

The human NSCLC cell lines A549, H1299, H1975, and H1650 were obtained from the American type Culture Collection. A549 and H1299 cells are EGFR wild type, while H1650 and H1975 harbor mutations in the EGFR tyrosine kinase-domain. H1650 cells contain a deletion in exon 19 (DelE746A750) and the H1975 cell line carries two missense mutations (L858R, T790M) in the EGFR gene [35]. Cells were grown in RPMI 1640 supplemented with 10% fetal bovine serum (FBS) (Euroclone; Milan, Italy) at 37 °C in 5% CO_2_/95% air. Cells were counted under a microscope (10× objective) using a Bright Line hemocytometer (Sigma-Aldrich; Milan, Italy) and cell viability was estimated using trypan blue staining. RIPA buffer (50 mM HEPES (pH 7.6), 150 mM NaCl, 10% glycerol, 1% Triton X-100, 1.5 mM MgCl_2_, 1 mM EGTA, 10 mM Na_4_P_2_O_7_, 100 mM NaF, 1 mM Na_3_VO_4_) plus protease inhibitors (Roche; Milan, Italy) were used to obtain cell lysates and protein concentration was determined using the Bradford assay (Bio-Rad; Milan, Italy). Conditioned medium was obtained as previously described [20].

### 4.6. Treatments

sEGFR and EGF recombinant proteins were purchased by PeproTech (Rocky Hill, NJ, USA) and were dissolved in sterile water as a stock solutions of 100 µg/mL. Both molecules were stored at −20 °C in tightly sealed sterile tubes and diluted, to the concentrations of 1 µg/mL for sEGFR and 100 ng/mL for EGF, in RPMI 1640 during each experiment.

### 4.7. Cell Migration Assay

Cell migration was evaluated using modified Boyden chambers as previously described [36]. Briefly, cells were treated with sEGFR (1 µg/mL) for 24, 48, and 72 h, followed by washing of 5 × 10^4^ cells with PBS, resuspended in serum-free medium and placed in the upper compartment. RPMI 1640 containing 0% or 10% FBS was added to the lower compartment. The chambers were separated by a polycarbonate filter (8 μm pore-size) that was pre-coated with collagen (50 µg/mL). After 6 h of incubation, the filters were recovered, cells on the upper surface mechanically removed, and cells migrated in the lower filter surface fixed with absolute ethanol and stained with DAPI. Cells were counted in a double-blind manner in five consecutive fields each with a fluorescent microscope. All experiments were performed three times.

### 4.8. Western Blot Analysis

Cell lysates were separated by SDS-PAGE (4%–20% Mini-Protean TGX precast gels; BioRad; Milan, Italy) and transferred to a nitrocellulose membrane. Membranes were probed with the specific antibody for 3 h at RT. Immunoblot analysis for sEGFR/EGFR status were performed by using two different antibodies against the N-terminal EGFR ECD: a mouse monoclonal antibody (Ab1_EGFR) kindly provided by Dr. Tagliabue (Fondazione IRCCS–Istituto Nazionale Tumori, Milan, Italy), and a goat polyclonal antibody (Ab2_EGFR) from R&D Systems (Milan, Italy). Cyclin D1 levels were determined by using a mouse monoclonal antibody (Santa Cruz Biotechnology; Santa Cruz, CA, USA). As a loading control, blots were stained using a mouse monoclonal antibody against β-Actin (Sigma-Aldrich; Milan, Italy).

### 4.9. Immunofluorescence and Microscopy Analysis

To identify the EGFR holoreceptor localization we used an immunostaining procedure. Briefly, cells were fixed using 8% paraformaldehyde diluted 1:2 in PBS for 10 min and permeabilized with 0.2% Triton X-100 in PBS for two minutes. Following a blocking step with 2% bovine serum albumin (BSA) (Sigma-Aldrich; Milan, Italy) and 20% fetal bovine serum (Euroclone; Milan, Italy) in PBS for 30 min, the antibody Ab1_EGFR, diluted 1:750 in PBS with BSA 2%, was applied for one hour. After washing with PBS, samples were incubated with FITC-conjugated anti-mouse secondary antibody (Invitrogen, Milan, Italy) for 40 min. DAPI (Sigma-Aldrich; Milan, Italy) was used to counterstain the cell nuclei. Coverslips were mounted with Vectashield (Vector, Burlingame, CA, USA) and immunofluorescence visualized in fluorescence microscope (Nikon, Melville, NY, USA).

## 5. Conclusions

In the present study, we observed a significant decrease of plasmatic sEGFR values in NSCLC patients when compared with control group (healthy population). Moreover, we investigated the effect of recombinant sEGFR on lung cancer cell lines, observing an anti-proliferative effect of this protein.

These data suggest that sEGFR is a circulating EGFR isoform with a protective role, representing a potential biomarker in NSCLC early-detection. However, the real impact of these data on daily clinical practice needs to be further investigated in larger prospective clinical trials.

## Supplementary Materials

Supplementary materials can be found at http://www.mdpi.com/1422-0067/16/08/19612/s1.
