# Supplementary material for: Preliminary Evidence on the Diagnostic and Molecular Role of Circulating Soluble EGFR in Non-Small Cell Lung Cancer
Source: Int J Mol Sci. 2015 Aug 19;16(8):19612–30. doi: 10.3390/ijms160819612 (PMC4581315; doi:10.3390/ijms160819612)
Supplement: Supplementary file 1 [file ijms-16-19612-s001.pdf]

Supplementary Information

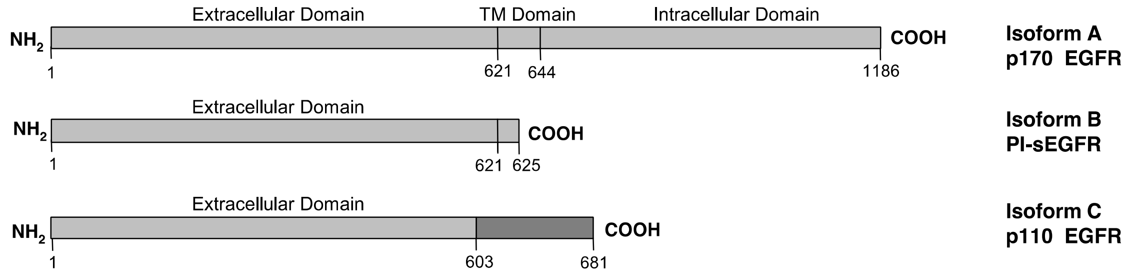

Figure S1. Diagrams representing specific EGFR isoforms. Isoform A correspond with the full-length EGFR (amino acids 1–1186) a transmembrane form having a molecular weight of 170 kDa. Isoform B and C represent both EGFR isoforms of about 110kDa, containing the same N-terminal portions and a different COOH-terminal. While Isoform B is called PI-sEGFR and arising from the proteolytic cleavage of EGFR holoreceptor between the amino acid residues 625–626, Isoform C (also called p110) has a 78 C-terminus unique amino acid sequence derived from EGFR gene alternative splicing. The C-terminal sequence of p110 is illustrated in dark gray.

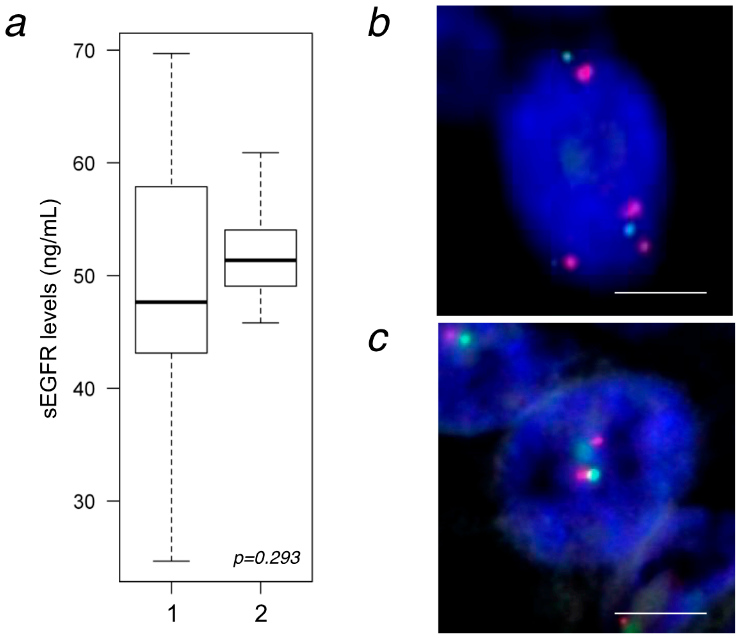

Figure S2. EGFR-copy number analysis and correlation with sEGFR levels in NSCLC patients. (**a**) Box plot depicts the sEGFR (ng/mL) levels in plasma from 30 patients having EGFR copy number wild type (1) and 7 patients with EGFR copy number amplification (2). Horizontal bold line, median value; the interquartile range is indicated above and below each bar; (**b**,**c**) Fluorescence in situ hybridization (FISH) for the EGFR gene expression in FFPE sections from lung tumors. Representative images of tumor samples with increased EGFR gene copy number (**b**) and tumors having wild type EGFR-copy number (**c**). Cells were counterstained with DAPI (blue) to label nuclei. SpectrumOrange-labeled probes hybridize to the entire EGFR gene region targeted, SpectrumGreen-labeled probes hybridize to the centromere of the chromosome appropriate for the gene-specific assay (chromosome 7; 7p11.1-q11.1). Magnification: 100×. Scale bar: 5μm
